# Supplementary material for: Genome-Wide Identification of Histone Deacetylases and Their Roles Related with Light Response in Tartary Buckwheat (Fagopyrum tataricum)
Source: Int J Mol Sci. 2023 Apr 30;24(9):8090. doi: 10.3390/ijms24098090 (PMC10179446; doi:10.3390/ijms24098090)
Supplement: Supplementary file 1 [file ijms-24-08090-s001.zip › ijms-2367275-supplementary.pdf]

Supplementary Materials

Table S1. Summary of primers used in this study.

Gene	Forward(5'-3')	Reverse(5'-3')
**For RT-qPCR**		
FtHDA2	ACCGGATTCTGTCGAGCAAG	TGCGTCCCCATTTTGAGGAA
FtHDA5	TCCCTTCGAGTCAACATGGC	CAGAATCAGAGCTCGGGCAT
FtHDA6-1	TTCTCTCCCCTGTCGGGAAT	CCTGCAGAAGCCTGACAGAA
FtHDA6-2	TGGAGATCAACCGCCCTTTC	CGCGAATGAGAGTGATCGGA
FtHDA8-1	AGGCTTTCTTGACGTCCTCG	ATTCGGAGAGTGAAGCTGGC
FtHDA8-2	TGGCGGAAAGGAACTGTGTT	GTATGTGCTTCATGGCTGCG
FtHDA9	GAGAACCCTGACAAACGCCT	AGCGTCGTCCATGTTGTGAT
FtHDA14	ATGCAGCGTCTTTTGTGCTG	ATGACCAGAGGCAGGAGCTA
FtHDA19	GTTCAGTCGGGGGATCTGTG	CACGTACAAGACTCGCCCAT
FtSRT1	AAGGACAGATTGCGGGACAG	AAGCTTCCCGCCATTACGAA
FtSRT2	ATGATGCAACGGTCTACGCA	AACGCTTACCGGTTGTTCCT
FtHDT1	TGACTGGAAACATGGGAGCG	GCTTTTGTTGCAGCTACCCC
FtHDT2	GAGCCAAGTAGTCCACCTTTCAC	CAGACAGAATCCCAATCACCAAT
FtHDT3	TCATGTGGCAACCCCATACC	CAAGAGCTGCAGGTAACGGA
FtH3	GAAATTCGCAAGTACCAGAAGAG	CCAACAAGGTATGCCTCAGC
FtACTIN7	CCGCCACTCAACACAATGTTATTAT	GAGTTATGAGCTTCCTGATGGACAA

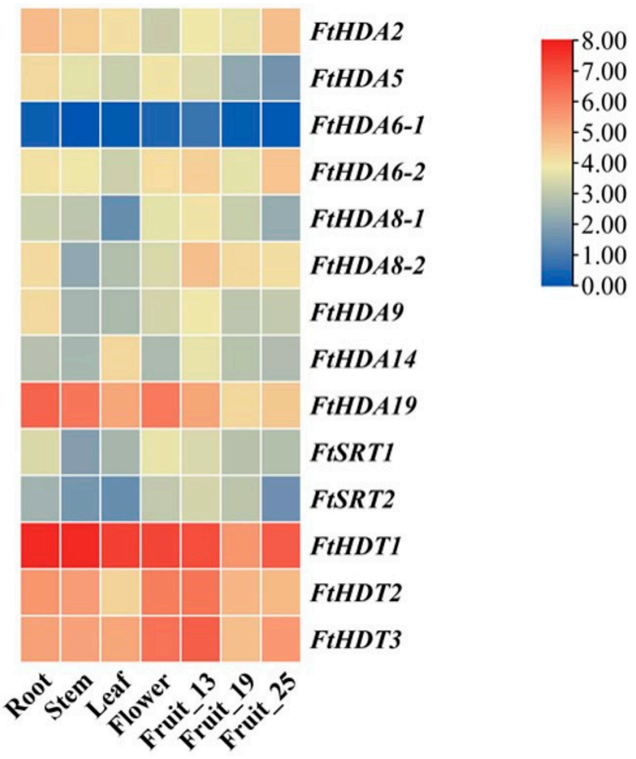

Figure S1. Expression patterns of 14 *HDAC* genes in diverse tissues of *F. tataricum*. RNA-seq data were downloaded from Tartary Buckwheat Database using gene IDs of 14 FtHDACs. The RPKM (Reads Per Kilobase of exon model per Million mapped reads) values were transformed to $\log_2$ (value + 1) and used for heatmap. The expression in diverse *F. tataricum* tissues was demonstrated, including root, stem, leaf, flower, fruit_13, fruit_19 and fruit_25.

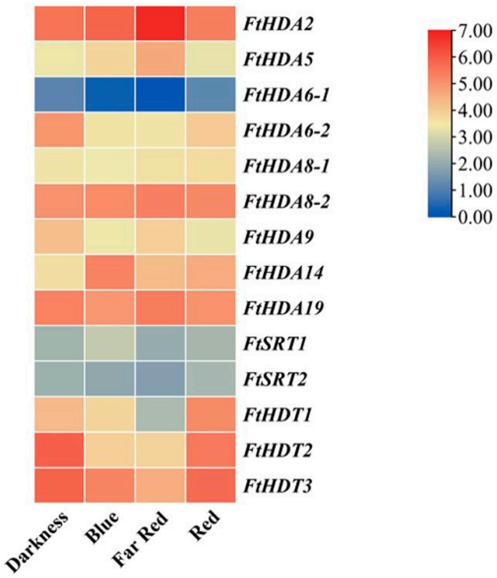

Figure S2. Expression profiles of 14 *FtHDAC* genes under different light wavelengths. The transcript profiles of *FtHDAC* genes in seedlings of *F. tataricum* under different light wavelengths (red light (670 nm), blue light (470 nm), and far-red light (735 nm)) were extracted from Zhang et al.[37]. The RPKM (Reads Per Kilobase of exon model per Million mapped reads) values were transformed to $\log_2$ (value + 1) and used for heatmap.

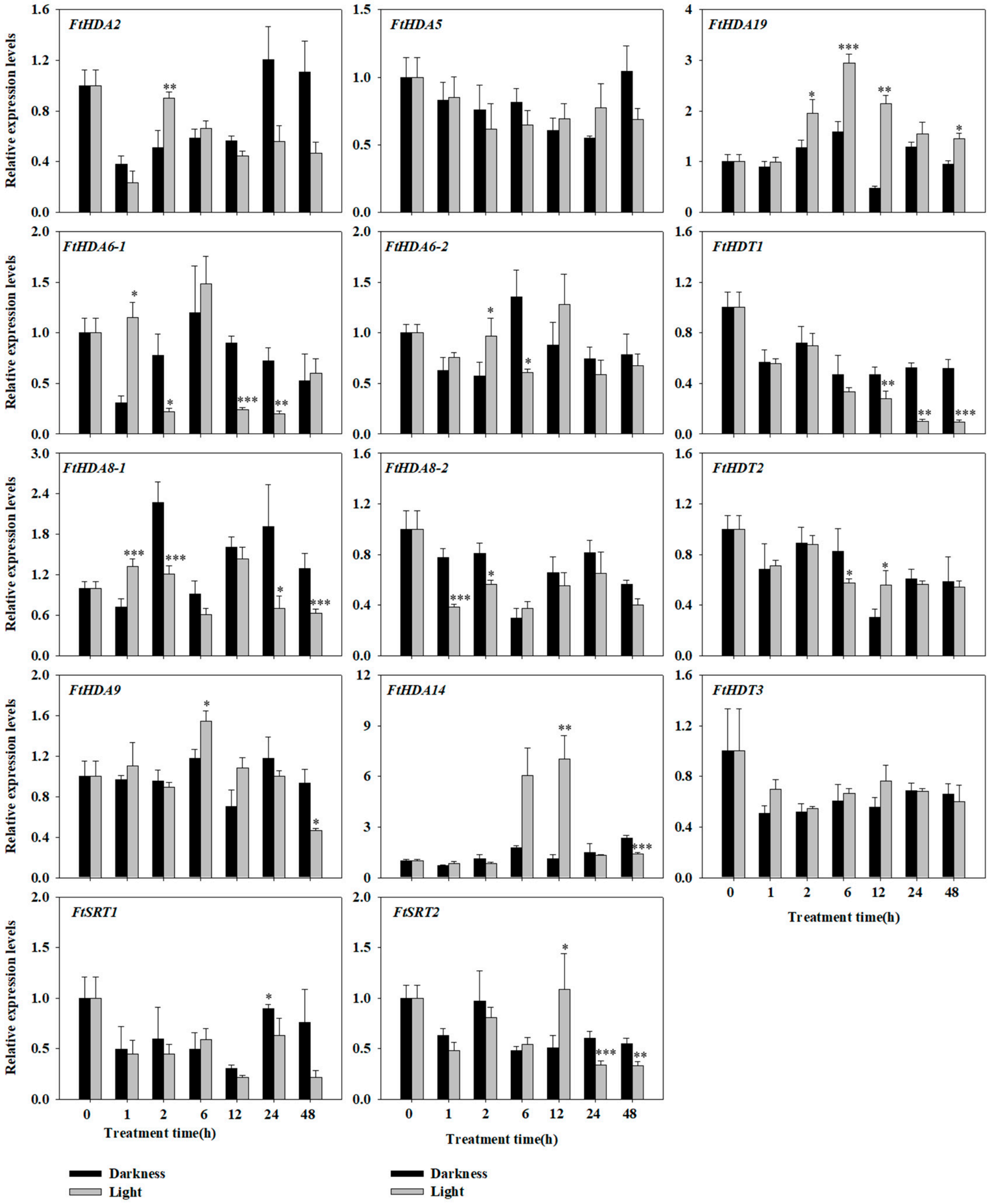

Figure S3. Expression profiles of 14 *FtHDAC* genes in response to light. *FtACTIN7* was used as reference gene. Expression levels of each gene were expressed as a ratio relative to that of untreated seedlings (Darkness 0 h), which was set as 1. Each data point represents a mean ± standard error (n=3). Asterisks above the bars indicate significant differences ($p<0.05$) among the treatments.
